# Occupational injuries among building construction workers in Addis Ababa, Ethiopia

**DOI:** 10.1186/s12995-016-0107-8

**Published:** 2016-04-11

**Authors:** Sebsibe Tadesse, Dagnachew Israel

**Affiliations:** Institute of Public Health, the University of Gondar, Gondar, Ethiopia; City Government of Addis Ababa Health Bureau, Addis Ababa, Ethiopia

**Keywords:** Construction industry, Occupational injuries, Workers, Workplace safety

## Abstract

**Background:**

Occupational injuries can pose direct costs, like suffering, loss of employment, disability and loss of productivity, and indirect costs on families and society. However, there is a dearth of studies clarifying the situation in most of Subsaharan African countries, like Ethiopia. The present study determined the prevalence of injury and associated factors among building construction employees in Addis Ababa, Ethiopia.

**Methods:**

An institutional-based cross-sectional study was conducted among building construction employees in Addis Ababa, Ethiopia from February to April 2015. Multi-stages sampling followed by simple random sampling techniques was used to select the study participants. The sample size of the study was 544. A pre-tested and structured questionnaire was used to collect data. Multivariable analyses were employed to see the effect of explanatory variables on injury.

**Results:**

The prevalence of injury among building construction employees was reported to be 38.3 % [95 % CI: (33.9, 42.7)] in the past 1 year. Use of personal protective equipments, work experience, khat chewing were factors significantly associated with injury.

**Conclusion:**

This is among the few studies describing construction health and safety in Ethiopia. In this study a relatively higher prevalence of injury was reported among building construction employees compared to other studies. If urgent interventions are not in place, the absence from work, loss of productivity and work-related illnesses, disabilities and fatalities will continue to be a major challenge of the construction industry in the future. Therefore, programs to mitigate the burden borne by construction-related injuries should focus on areas, such as provision of safety trainings, promoting use of PPE and monitoring substance abuse in workplace.

## Background

The World Health Organization defines occupational injury as an epidemic problem in the field of public health in developing countries [1, 2]. The human suffering caused by the injuries is hurtful to the employee, the employer and society [3–5]. According to the International Labor Organization there are 270 million occupational accidents causing 2 million deaths annually [6]. In the United States the cost of occupational injuries was $177.2 billion, and 35 million working days were lost annually [3]. The construction industry is responsible for more than half of all occupational injuries and deaths worldwide [7]. It is widely recognized as having high accident rates which result in absence from work, loss of productivity, permanent disabilities and even fatalities [8]. The estimated direct and indirect costs of fatal and nonfatal construction injuries totaled about $13 billion annually. The medical expenses of nonfatal injuries alone cost more than $1.36 billion annually [9].

Construction is a sector that has very specific hazards, like work at heights, work with power tools, more than one trade and more than one employer/contractor working on a single site with lack of coordination, working in the outdoor elements, work with power tools, contractual work as opposed to permanent employment, lack of standards or regulations among workers in terms of expertise in their trade and training standards, less regulation and enforcement than other sectors. Studies reveal that there are various factors that are significantly associated with occupational injury. These factors include lack of health and safety programs, young workers, male sex [10], lack of formal education [11], smoking [12], sleeping problems [13], lack of physical exercise [14], frequent alcohol consumption [12], extended work hours [15], night work [15], physically demanding work [16], low job experience [15], and non-use of personal protective equipment [17].

The impact of occupational health and safety hazards faced by construction workers in developing countries is 10 to 20 times higher than those in industrial countries [18]. In Ethiopia information regarding construction injuries is rare, and very limited attempts have been made to investigate the prevalence and associated factors [19]. This paper presents the findings of a study which investigated prevalence and factors associated with occupational injuries among building construction workers in Addis Ababa, Ethiopia. The information could help in designing appropriate preventive and control strategies.

## Methods

### Study design, area and period

A construction site-based cross-sectional study was conducted to assess prevalence and factors associated with occupational injuries among building construction workers in Addis Abba, the capital city of Ethiopia from February to April 2015.

### Participants

All employees who were directly involved in the process of construction in the last 1 year were included in the study until the required sample size was obtained. Workers who were absent from work for any reason during the time of data collection were excluded from the study.

### Survey tool

A pre-tested and structured interview questionnaire was used to collect the data. The questionnaire contained detailed information on socio-demographic, behavioral and environmental factors that could have association with injuries. The respondents were asked the question stated as, “Have you ever had any physical injury resulting from an accident in the course of construction work in the past 1 year?” to determine prevalence of injury. A generic job satisfaction scale was used to assess workers status of job satisfaction. The respondents were also asked a close ended question to determine the main causes of injuries as perceived by them: “What are the main causes of injury at this workplace? 1. Lack of awareness 2. Poor working conditions 3. Lack of PPE 4. Others”.

### Sample size calculation

Epi info version 7 was used to determine the sample size of 544 by taking 6999 total population of construction workers, 38.7 % expected proportion of injury [19], 5 % confidence limit, 95 % confidence level, 5 % non-response rate, 1.5 design effect.

### Sampling procedure

The multi-stage sampling technique was used to select the study participants. In the first stage, 4 condominium construction sites were randomly selected by the lottery method from 8 sites in the Addis Ababa city administration. In the second stage, the total of 544 samples was proportionally allocated to each selected sites (i.e. 82 to site 1 (N1 = 546), 128 to site 2 (N2 = 840), 148 to site 3 (N3 = 970), and 186 to site 4 (N4 = 1227)). The participants were drawn from the site’s list of workers using simple random sampling. Three trained degree holders participated in the data collection processes.

### Data quality control

The training of data collectors and supervisors emphasized issues such as the data collection instrument, field methods, inclusion–exclusion criteria and record keeping. The investigators coordinated the interview process, and spot-checked and reviewed the completed questionnaire on a daily basis to ensure the completeness and consistency of the data collected. The interview questionnaire was pre-tested on 20 respondents in order to identify potential problem areas, unanticipated interpretations and cultural objections to any of the questions. Based on the pre-test results, the questionnaire was adjusted contextually.

### Data management and statistical analyses

Data entered and cleaned using Epi info version 7 statistical software were analyzed on SPSS version 20. Frequency distribution, mean, standard deviation and percentage, were employed for most variables. All independent variables were fitted separately into a bivariate logistic model to evaluate the degree of association with injuries. Then, variables with a *p*-value < 0.20 were exported to multivariable logistic regression model to control confounders. The odds ratio (OR) with a 95 % confidence interval (CI) was used to test the statistical significance of variables. Only statistically significant variables were presented.

### Operational definitions

#### Occupational injury

Any physical injury resulting from an accident in the course of construction work in the past 1 year prior to this study.

#### Job satisfaction

The employee was considered as satisfied with job when his/her sum of generic job satisfaction scale score was 32 or above [20].

#### Personal Protective Equipment (PPE)

Utilization of specialized clothing or equipment worn by employees for protection against health and safety hazards. Workers were classified as *those who used PPE* when they were observed wearing the PPE that were necessary to be worn during a particular activity.

### Permanent employee

Any contract of employment between employee and employer concluded for an indefinite period [21].

### Temporary employee

Any employment contract between employee and employer made for defined period [21].

### Cigarette smoker

An employee who was smoking one cigarette a day for at least 1 year [22].

### Alcohol drinker

An employee who drinks at least five drinks per week for men and two drinks per week for women for at least 1 year [22].

### Khat chewer

An employee chewing khat (a mild psychoactive substance) three times a week for at least 1 year [22].

### Ethical considerations

The study protocol was reviewed and approved by the Institutional Review Board of the University of Gondar via the Institute of Public Health. Permission was obtained from city government of Addis Ababa Social Affair Office prior to data collection. Study participants were interviewed after informed written consent was obtained. They were also informed that their participation was voluntary and that they could withdraw from the interview at any time without consequences. The participants were assured that their responses would be treated confidentially through the use of strict coding measures. Finally, safety education was given to workers who reported injuries. They were told to avoid unsafe acts, to use PPE and to follow safety rules.

## Results

### Socio-demographic characteristics

A total of 504 employees completed the questionnaire making response rate 92.6 %, of whom 62.9 % were males. The majority, 89.5 %, of the employees belonged to the age group of 18–35 years. Half, 50.4 %, attended secondary and higher education. Regarding religion 72.4 % of the employees were Christian. The single were 58.3 %. The majority, 80.2 %, had a monthly salary of Birr 1000–3000 (Table 1).

**Table 1:** Socio-demographic characteristics of building construction employees in Addis Ababa, Ethiopia, 2015

Variables	Number	Percent
Sex		
Male	317	62.9
Female	187	37.1
Age (in years)		
18–35	451	89.5
> 35	53	10.5
Marital Status		
Single	294	58.3
Married	176	34.9
Widowed/divorced	34	6.8
Educational status		
Illiterate	30	6.0
Primary	220	43.6
Secondary and above	254	50.4
Religion		
Christian	365	72.4
Muslim	137	27.2
Other	2	0.4
Monthly salary (in US $)		
50–150	404	80.2
> 150	100	19.8

### Workplace and behavioral characteristics

Three-fourths, 75.8 %, of the participants were temporary employees. The majority, 84.7 %, served for less than 2 years. Regarding hours spent on work 91.9 % of the employees had worked for ≤8 h per day. More than three-fourths, 77.8 %, of them were satisfied with their job. The majority, 76.6 %, did not use PPE. Eighty four percent did not attend any kind of workplace safety training. The majority, 59.1, 73.4 and 91.3 %, of the employees didn’t drink alcohol, chew khat and smoke cigarette, respectively (Table 2).

**Table 2:** Workplace and behavioral characteristics of building construction employees in Addis Ababa, Ethiopia, 2015

Variables	Number	Percent
Employment pattern		
Permanent	122	24.2
Temporary	382	75.8
Work experience (in years)		
≤ 2	427	84.7
> 2	77	15.3
Working hours per day		
≤ 8	463	91.9
> 8	41	8.1
Job satisfaction		
Satisfied	392	77.8
Dissatisfied	112	22.2
Safety training		
Yes	82	16.3
No	422	83.7
Use PPE		
Yes	118	23.4
No	386	76.6
Drink alcohol		
Yes	206	40.9
No	298	59.1
Smoke cigarette		
Yes	44	8.7
No	460	91.3
Chew khat		
Yes	134	26.6
No	370	73.4

### Prevalence of Injury

**Fig. 1:**
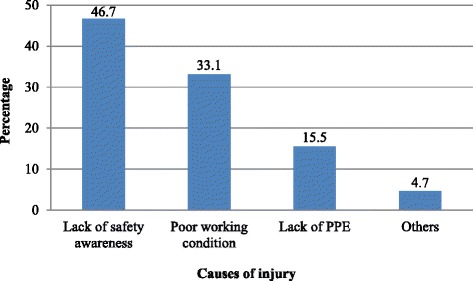
Causes of injuries reported by employees

The prevalence of injury among building construction employees was reported to be 38.3 % [95 % CI: (33.9, 42.7)] in the past 1 year, of whom 62.2 % were males. The majority, 79.3 and 83.9 %, served for less or equal to 2 years and did not use PPE, respectively. The common types of injuries were 66.3 % cutting and 28.5 % falling. Nearly half, 46.6 %, of the incidents were leg injuries followed by 43.5 % finger/hand. The major cause of injuries was lack of safety awareness, 46.7 % (Fig. 1).

### Factors associated with injury

Table 3 presents factors which remained statistically significant in the bivariate and multivariable logistic regression analyses. In this study the independent predictors of injury on the multivariable analysis include use of PPE [AOR: 0.4, 95 % CI: (0.2, 0.7)], work experience [AOR: 0.4, 95 % CI: (0.2, 0.7)], khat chewing [AOR: 2.6, 95 % CI: (1.6, 4.2)] (Table 3).

**Table 3:** Factors associated with injuries among building construction employees in Addis Ababa, Ethiopia, 2015

Variables	Injuries		Crude OR (95 % CI)	Adjusted^a^ OR (95 % CI)
	Yes	No		
Use PPE				
Yes	31	91	0.5 (0.3, 0.7)	0.4 (0.2, 0.7)
No	162	220	1	1
Work experience (in years)				
≤ 2	153	274	0.5 (0.3, 0.8)	0.4 (0.2, 0.7)
> 2	40	37	1	1
Chew khat				
Yes	70	64	2.2 (1.5, 3.3)	2.6 (1.6, 4.2)
No	123	247	1	1

^a^ The multivariable model was adjusted for age, sex, educational status, marital status, employment pattern, monthly income, cigarette smoking, alcohol drinking, job satisfaction, working hours per day and safety training

## Discussion

Industrial safety and health problems are becoming major challenges in Ethiopia because of low occupational hazards awareness, lack of workplace safety and health policy, and inefficient safety management systems. Due to these factors employers, workers and the government are incurring measurable and immeasurable costs. Injuries remain the major occupational health problem among construction employees [8, 9]. In this study the prevalence of injury among the employees was 38.3 % [95 % CI: (33.9, 42.7)]. This finding is in line with a study from Ethiopia (38.7 %) [19], and higher than that of studies from Egypt (18.4 %) [23] and India (22.9 %) [24]. The discrepancy could be due to methodological differences, like study populations, methods of data collection and workplace conditions, like employees’ level of awareness of hazard control and disease prevention and accessibility of workplace safety services. Emphasis on preventive measures, such as short and long-term training as well as encouragement to use safety tools can effectively decrease the prevalence of occupational injuries [7].

This study identified important predictors influencing the occurrence of occupational injury. The odds of injuries among employees who used PPE were 60 % less compared to those who did not. Use of PPE is one of the important measures to safeguard workers from exposure to occupational hazards, especially in developing countries where conventional occupational safety control measures remain a challenge to implement [25–27]. PPE is the lowest measure in the hierarchy of hazard control that works because it depends on workers’ behavior [28]. Engineering controls, substitution and administrative controls are more effective methods that do not depend on workers’ behavior. In this study more than three-fourths of the employees did not use PPE during work. This may signify that there was poor provision of PPE from employers, and lack of awareness about its importance by the workers. As a recommendation, it is imperative that safety programs need to pay more attention to provision and use of PPE.

Another important finding of this study was that the odds of injuries among employees who served for less or equal to 2 years were 60 % less compared to those who served for more than 2 years. The possible explanation for this may be that those employees who served for greater than 2 years could be accustomed to the work environment and developed false consciousness of safety which drive them not to comply with safety precautions including proper use of PPE. It might also be due to the fact that the lack of safety awareness programs in the workplaces, poor working conditions and lack of PPE, which were described in this study, could influence employees’ experience of injuries during their longer stays. Finally, there could be a healthy worker effect, since absent workers were not included in the study and may have left this workforce due to injury.

The odds of injuries among employees who chewed khat were about three times more compared to those who did not. This might be due to the fact that abuse of mind altering substances, like khat is likely to cause a change in the behavior and impair workers concentration and performance. A high blood level of such substances while at work will endanger both safety and efficiency, and be the cause of increased likelihood of mistakes, poor decision making and errors in judgment. As the result of this fact the industries’ safety policy should consider control of substance abuse at workplace.

There are several limitations of this study that should be noted. Social desirability bias is a potential limitation in self-reported studies like this one, in that employees might report more socially acceptable responses than their actual day to day practice. In this study occupational injury is defined as any physical injury resulting from an accident in the course of construction work in the past 1 year prior to this study. Therefore, further studies need to be conducted to explain the nature of injury by its severity. As this is a cross-sectional study, the cause-effect relationship is not established between the different independent variables and injury. Moreover, injury status of the 40 workers who were selected but did not complete the questionnaire was not known. The prevalence is likely to be higher with the exclusion of injured workers –“healthy worker effect”.

## Conclusions

This is among the few studies describing construction health and safety in Ethiopia. In this study a relatively higher prevalence of injury was reported among building construction employees compared to other studies. If urgent interventions are not in place, the absence from work, loss of productivity and work-related illnesses, disabilities and fatalities will continue to be a major challenge of the construction industry in the future. Therefore, programs to mitigate the burden borne by construction-related injuries should focus on areas, such as provision of safety trainings, promoting use of PPE and monitoring substance abuse in workplace.

## References

[1] Karvonen M. Epidemiology in the context of occupational health. In: Karvonen M and Mikheev MI. Epidemiology of Occupational Health. WHO. Copenhagen; WHO Regional Office Office for Europe: 1986. 1–15.

[2] Hamalainen P (2009). The effect of globalization on occupational accidents. Saf Sci.

[3] Larsson TJ, Field B (2002). The distribution of occupational injury risks in the Victorian construction industry. Saf Sci.

[4] Lowery JT, Glazner J, Borgerding JA, Bondy J, Lezotte DC, Kreiss K (2000). Analysis of construction injury burden by type of work. Am J Ind Med.

[5] Moradinazar M, Kurd N, Farhadi R, Amee V, Najafi F. Epidemiology of Work-Related Injuries Among Construction Workers of Ilam (Western Iran) During 2006 – 2009. Iran Red Crescent Med J. 2013;15(10):e8011. 10.5812/ircmj.8011 PMC3950784 24693372

[6] International Labor Organization. Work-related fatalities reach 2 million annually 2002. Available from: http://www.nieuwsbank.nl/en/2002/05/24/K016.htm. Accessed March 20 2015.

[7] Lopez-valcarcel A (2001). Occupational safety and health in the construction work. Afr Newsl Occup Health Safety.

[8] Eid AH, Sewefy AZ (2009). Health Hazards and safety. J Egypt Med Assoc.

[9] CPWR (2008). The center for construction research and training. Construction chart book.

[10] Zewdie A (2009). Determinants of occupational injury: a case control study among textile factory workers in Amhara regional state, Ethiopia. J Trop Med.

[11] Kunar BM, Bhattacherjee A, Chau N (2008). Relationships of job hazards, lack of knowledge, alcohol use, health status and risk taking behavior to work injury of coal miners: a case-control study in India. J Occup Health.

[12] Bhattacherjee A, Chau N, Sierra CO, Legras B, Benamghar L (2003). Relationships of job and some individual characteristics to occupational injuries in employed people: a community-based study. J Occup Health.

[13] Salminen S, Oksanen T, Vahtera J, Sallinen M, Harma M (2010). Sleep disturbances as a predictor of occupational injuries among public sector workers. J Sleep Res.

[14] Gauchard GC, Chau N, Touron C, Benamghar L, Dehaene D (2003). Individual characteristics in occupational accidents due to imbalance: a casecontrol study of the employees of a railway company. Occup Environ Med.

[15] Dembe AE, Erickson JB, Delbos RG, Banks SM (2005). The impact of overtime and long work hours on occupational injuries and illnesses: new evidence from the United States. Occup Environ Med.

[16] Smith PM, Mustard C (2004). Examining the associations between physical work demands and work injury rates between men and women in Ontario, 1990–2000. Occup Environ Med.

[17] Kumar SG, Rathnakar U, Harsha KH (2010). Epidemiology of accidents in tile factories of mangalore city in karnataka. Indian J Community Med.

[18] Dong X (2005). Long work hours, work scheduling and work-related injuries among construction workers in the United States. Scand J Work Environ Health.

[19] Molla M, Alemu K, Kebede G, Rai H, Worku W (2013). Occupational injuries among building construction workers in Gondar City, Ethiopia. Occup Med Health Aff.

[20] Scott Macdonald MP (1997). The Generic Job Satisfaction Scale: Scale Development and Its Correlates.

[21] Ministry of Labor and Social Affairs (2003). Labour proclamation No.377/2003.

[22] Melchior M, Niedhammer I, Berkman LF, Goldberg M (2003). Do psychosocial work factors and social relations exert independent effects on sickness absence? A six year prospective study of the GAZEL (France) cohort. J Epidemiol Community Health.

[23] Alazab RM (2004). Work-related diseases and occupational injuries among workers in the construction industry. Afr Newsl Occup Health Safety.

[24] Shah CK, Mehta H (2009). Study of injuries among construction workers in Ahmedabad City, Gujarat. Indian J Practicing Doctor.

[25] Malik N, Mean AA, Pasha TS, Akhtar S, AIi T (2010). Role of hazard control measures in occupational health and safety in the textile industry of Pakistan. Pak J Agric Sci.

[26] Kamal A, Sayed M, Massoud A (2007). Usage of personal protective devices among Egyptian industrial workers. Am J Ind Med.

[27] Akintayo WL (2013). Knowledge, attitude and practice on the use of personal protective equipment by traditional resist Fabrics workers in Abeokuta, Nigeria. Kuwait Rev Chapter Arab J Bus Manag Rev.

[28] Jaiswal A. Case-control study among carpet thread factory workers in Uttar Pradesh, India: Occupational injury and its deteriorating factors. Glob J Hum Soc Sci Hist Anthr. 2012;12(10). ISSN: 2249–460x.

